# A Review of the Therapeutic Challenges and Novel Treatment Options in Mitigating Invasive Mycosis Associated with *Aspergillus*, *Candida*, *Cryptococcus*, and *Pneumocystis*

**DOI:** 10.3390/jof12090659

**Published:** 2026-09-02

**Authors:** Elaine Meade, Mark Slattery, Mary Garvey

**Affiliations:** 1 Department of Life Science and Pharmacy, Atlantic Technological University, Ash Lane, F91 YW50 Sligo, Ireland; 2 Mark Anthony Slattery, Veterinary Surgeon, F92 E619 Sligo, Ireland

**Keywords:** mycosis, invasive, resistance, undiagnosed, priority pathogens, mortality

## Abstract

Globally, invasive fungal diseases are increasing in incidence and are associated with high rates of mortality. A lack of effective diagnostic protocols, antifungal resistance and poor antifungal stewardship contribute to negative outcomes. *Aspergillus*, *Candida*, *Cryptococcus*, and *Pneumocystis* are the most common pathogens associated with fatal fungal diseases. These species are responsible for 90% of reported invasive fungal deaths globally. Fungal species employ a diverse and effective array of virulence factors to sabotage, evade, and manipulate host immune systems, allowing for colonisation and dissemination in vivo. Invasive fungal infections are clinically challenging, as they penetrate organs and deep tissues, contributing to the aetiology of respiratory tract, endocarditis, meningitis, and medical device infections and resulting in false negative diagnostic attempts. The 2025 WHO report outlines a lack of diagnostic and therapeutic options available, especially in low-to-middle-income countries. Geographical and economic factors impact fungal disease and mortality rates. There is an urgent need for new approaches to combat fungal diseases clinically with improved biocompatibility profiles. This timely review discusses the prevalent invasive fungal species, namely, *Candida*, *Aspergillus*, *Cryptococcus*, and *Pneumocystis,* which have high mortality rates, as identified by the WHO priority pathogen list, and discusses recent advances in the treatment of fungal diseases. This dissemination of information is an important aspect of the United Nations Sustainable Development Goals (SDGs) and One Health.

## 1. Introduction

The publication of the World Health Organisation (WHO) fungal priority pathogen list aimed to highlight the increasing risk of mycosis and invasive fungal diseases (IFDs), where antimicrobial resistance (AMR) is a major contributor to mortality. AMR and multidrug resistance (MDR) are evident in many fungal pathogens isolated from clinical settings, with resistance demonstrated across all five antifungal therapy classes (azoles, allylamines, echinocandins, pyrimidines, and polyenes) [1]. The Center for Disease Control (CDC) states that fungal diseases (mycosis) result in 130,000 hospitalisations and 7300 deaths annually in the United States (US), with data from 2023 showing increasing rates of mortality from mycosis globally [2]. The prevalence of fungal diseases and associated mortality is underestimated, however, as demonstrated by recent studies [3]. The findings of Denning (2024) show mortality rates of ca. 85% for invasive aspergillosis (IA) in cancer, intensive care unit (ICU) and chronic obstructive pulmonary disease (COPD) patients, ca. 64% for invasive candidiasis, and ca. 76% for Cryptococcal meningitis (CM), with 3.8 million deaths from IFDs reported annually [3]. The findings of the Global Action For Fungal Infections (GAFFI) group detail a prevalence of 6.55 million cases of fungal diseases globally and 3.75 million deaths yearly, with a mortality of ca. 57% [4]. The fungal death rate is therefore greater than that of malaria, breast cancer and tuberculosis (TB) [4]. As heterotrophs, fungi are abundant in nature, playing an essential role in carbon cycling, decay and decomposition of dead organic matter and maintenance of ecosystems [5]. Typically, fungi thrive in ambient temperatures, with the human body temperature of 37 °C being unfavourable for fungal colonisation. Consequently, there are ca. 300 fungal species associated with human infection, such as superficial or invasive fungal infection (IFI), of which *Aspergillus*, *Candida*, *Pneumocystis* and *Cryptococcus* (Table 1) are the most common clinically relevant invasive pathogens in immunocompromised (IC) patients [6]. The clinical considerations for the drug resistance and mortality rates associated with these fungal pathogens are outlined in Table 1. The thermally dimorphic fungi *Histoplasma*, *Blastomyces*, *Coccidioides* and *Paracoccidioides* cause underdiagnosed fungal diseases (severe pulmonary diseases and IFIs) in immunocompetent persons and also have high mortality rates [7]. Superficial mycosis is a fungal infection of the skin, hair, and nails caused by dermatophyte species, including *Microsporum*, *Trichophyton*, and *Epidermophyton*, as well as other fungal species such as *Malassezia furfur* and *Candida* [8]. IFIs occur in vivo with fungal bloodstream infections (BSIs) and invasion of tissues and organs, and they are categorised as serious, deep, deep-seated, disseminated, and systemic fungal infections [9]. *Aspergillus*, a species of filamentous fungi, is commonly associated with the aetiology of lung diseases, including allergic bronchopulmonary aspergillosis, hypersensitivity, aspergilloma and invasive pulmonary aspergillosis [10]. Cases of MDR *Candidozyma auris* (formerly *Candida auris*) are increasing annually, with over 1346 cases of infection reported by the European Union (EU) in the 2024 *C. auris* survey [11]. However, the true number of cases is likely underestimated due to a lack of systematic surveillance [11]. As a WHO critical pathogen, *C. auris* is associated with BSIs and a mortality rate between 30% and 72% [12]. The increasing prevalence of mycosis is associated with an increasing population of IC and critical care patients [13]. Risk factors for mycosis include cancer, especially for autoimmune and AIDS patients; medical interventions/surgical procedures and medical devices; the presence of co-morbidities, e.g., diabetes, asthma, and COPD; and infectious diseases, e.g., TB. For example, Purschke et al. (2025) determined that IFIs in rheumatoid arthritis patients are rare but life-threatening when present [14]. Endogenous pathogens, for example, *C. albicans* and *C. glabrata,* are part of the human microbiota in the gastrointestinal tract (GIT), and abdominal surgery is associated with candida BSIs caused by these species (52.7% and 55.5%, respectively) [13]. The emergence of fungal diseases is also impacted by climate change, causing the migration and increased virulence of otherwise endemic fungi [15]. Climate change may support the geographical expansion and transmission of species including pathogenic *Coccidioides*, *Histoplasma*, and *Blastomyces* [7]. Interestingly, the studies by Jabrodini et al. (2024) show that the mandatory use of personal protective equipment (PPE) during the COVID-19 pandemic increased the rate of cutaneous infection due to disruption of skin integrity and altered skin microbiota [16]. Moreover, there is a disparity in the prevalence and mortality rates of IFIs in high-income countries (HICs) and low-to-middle-income countries (LMICs). For example, the mortality of *C*. *neoformans*-mediated meningitis is estimated at 70% in LMICs compared to 20–30% in HICs [17]. Additionally, there are no vaccines approved for fungal diseases due to their complex biology and immune-evading mechanisms. The high levels of AMR and complete lack of fungal vaccines highlight the need for improved diagnostic protocols, prophylactic measures and effective antifungal stewardship to reduce fungal morbidity and mortality. This important review discusses the prevalent invasive fungal species identified by the WHO as having high mortality rates, which is an important aspect of the United Nations Sustainable Development Goals (SDGs) and One Health. Candidiasis, aspergillosis, cryptococcosis, and pneumocystis are responsible for ca. 90% of reported invasive fungal deaths [18] and, as such, are the topic of this review. This dissemination of information and awareness of fungal infectious diseases is an important aspect of SDG topic 3 (ensure health and well-being) for the prevention of neonatal and tropical deaths by 2030 [19].

**Table 1:** The most common fungal pathogens present in the aetiology of human diseases and clinical considerations.

Fungal Pathogen	Clinical Considerations	Morbidity	Mortality Rate	Reference
*Aspergillus*—*A. fumigatus*	Azole and echinocandins resistance, cystic fibrosis, COPD, *Cytomegalovirus*, HCT recipients, TB and transplant patients at risk	IFI, sepsis, hypersensitivity	30–88% in ICU patients, ca. 60% in HCT recipients, 31–36% six-week mortality for invasive aspergillosis	[20,21,22]
*Candida—C. auris*, *C. ablicans*, *non-ablicans Candida*	Abdominal surgery, tumour and IC patients at risk, medical devices, HCT and CAR-T cell treatment patients at risk, MDR to echinocandins, azoles, and pyrimidine analogue (flucytosine)	Fungal BSIs, i.e., candidemia, IFIs, sepsis	Candidemia 30-day mortality of 42%, 55–60% for invasive *C. tropicalis*, 30–72% for *C. auris* hospital patients and 83% in cases of COVID-19 co-infection	[13,21,23,24]
*Cryptococcus*—*C. gattii*, *C. neoformans*	Advanced HIV patients at risk, caspofungin resistant, *C. neoformans* co-infection with TB	Pulmonary cryptococcosis, HIV-associated cryptococcal meningitis, *C. gattii* is associated with neurological sequelae and immune reconstitution inflammatory syndrome	*C. gattii* 10–43%, *C. neoformans* 20–61% in HIV patients and 8–50% in non-HIV patients, *C*. *neoformans*-mediated meningitis mortality of ca. 70% in LMICs	[17,25]
*Pneumocystis*—*P. jirovecii*	HIV, *Cytomegalovirus* patients, kidney transplant and vasculitis patients at risk, resistance to polyenes (AMP B) and azoles, horizontal transmission	*Pneumocystis* pneumonia (PCP), sepsis	31.5% in HIV IC patients and 46.6% in non-HIV IC patients	[3,23]

## 2. Clinically Relevant Invasive Fungi

The publication of the WHO FPPL in 2022 highlighted the global threat of fungal diseases. More recently, GAFFI reported an alarming fungal mortality rate, with many cases of IFDs going undiagnosed [4]. Moreover, antifungal resistance (AFR) contributes to increasing fungal mortality and poor clinical outcomes [26]. Fungal diagnostic tools suffer from many pitfalls, hindering their application and contributing to false negative results. An excellent review of fungal diagnostics is provided elsewhere [27]. The issue of poor diagnostics is compounded by a lack of AFR surveillance programmes, susceptibility testing, funding and fungal experts in clinical settings [22]. The WHO issued a report in 2025 detailing the critical lack of therapeutic and diagnostic tools for IFIs and highlighting the need for innovative research and development of diagnostic methods that can be accessed in LMICs [28]. The studies by Meade et al. (2024) describe the AFR profiles of *C. albicans*, *C. glabrata*, *C. tropicalis*, *C. krusei*, *C. parapsilosis*, and *C. neoformans* to AMP B, fluconazole, caspofungin, and flucytosine [29]. AMR in fungal species is a One Health issue, with ca. 99% of azoles, equating to 10,000 tonnes, used for agricultural, veterinary, and industrial chemicals in cosmetic and hygiene products in the EU [30]. An excellent review of AFR and the One Health Framework is provided elsewhere by Roohi et al. (2026) [30]. IFIs are problematic clinically as they penetrate organs and deep tissues, contributing to the aetiology of respiratory tract, endocarditis, meningitis, and medical device infections and often resulting in false negative blood culture diagnostic attempts [9]. Studies show that liver cirrhosis in patients reduces humoral and cellular immunity and increases the risk of IFIs [31]. Historically, neutropenia in patients for ca. 7 days with the administration of antibiotic therapy was considered a prerequisite for antifungal therapy. This application of antifungal therapy is clinically termed empirical antifungal therapy.

There are increasing rates of *Candida* BSIs [2] or invasive candidiasis annually, with a mortality rate of ca. 60% [22]. Furthermore, it is predicted that ca. 50% of invasive *Candida* infections go undiagnosed or undetected, meaning prevalence rates are greatly underestimated [32,33]. Importantly, the candidemia/intra-abdominal candidiasis in European ICU project (EUCANDICU), assessing candidemia in ICU patients in nine EU countries, reports an incidence of 7.07 cases per 1000 ICU admissions and a 30-day mortality of 42%, with *C. albicans* causative of 57%, *C. glabrata* of 21% and *C. parapsilosis* of 13% of cases [32]. In ICU patients, *Candida* is associated with candidemia BSIs and deep-seated candidiasis, which are commonly associated with the intra-abdominal area and peritonitis due to movement from the GIT [13]. The European Conference on Infections in Leukaemia (ECIL) guidelines suggest the administration of caspofungin (50 mg/day following 70 mg on day 1) or liposomal amphotericin B (AMPB) at 3 mg/kg for candidemia [33]. Notably, there is an increasing global prevalence of azole resistance in *C. albicans* and non-*Candida albicans* species, including *C. auris* [22]. Studies have identified *C. albicans* isolates with resistance to many azoles, including fluconazole, itraconazole, ketoconazole, voriconazole, and posaconazole [34]. When used as a monotherapy for urinary candidiasis, flucytosine resulted in 22% of strains developing resistance [24], highlighting the ease of resistance development to this antifungal. A combined antifungal approach, however, improves mortality associated with *Candida* meningitis in neonatal central nervous system (CNS) infections [24]. A combined therapeutic approach has a multi-hit effect, which overwhelms resistance mechanisms; for example, pore formation associated with AMPB allows flucytosine to enter cells. Studies report resistance to fluconazole, AMPB, and echinocandin in 90%, 30% and <5% of *C. auris* isolates, as determined using CDC antifungal breakpoints [2,35]. Resistance to fluconazole is alarming, as this therapeutic is better tolerated by infant patients [29]. Additionally, fluconazole is a commonly used antifungal agent.

**Figure 1:**
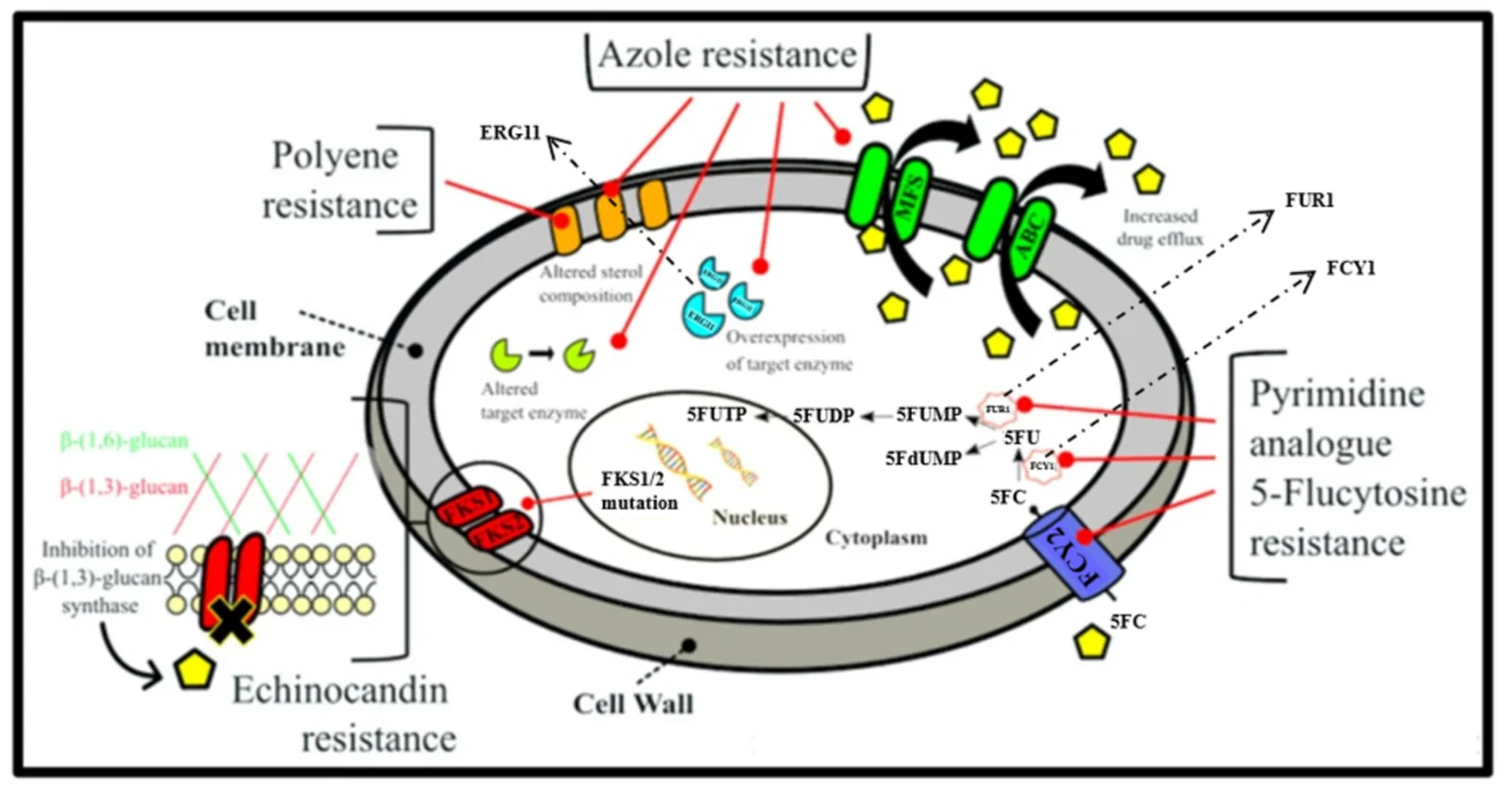
Major resistance mechanisms of fungal species promoting MDR in priority pathogens. Echinocandin resistance is associated with mutations in *FKS1/FKS2*, which alter β-(1,3)-glucan synthase and reduce inhibition of fungal cell wall synthesis. Polyene resistance can result from alterations in membrane sterol composition, reducing polyene binding to membrane sterols and subsequent membrane disruption. Azole resistance can arise through alterations or overexpression of target enzymes involved in ergosterol biosynthesis, together with increased drug efflux, reducing intracellular drug accumulation and activity. 5-Flucytosine (5FC) resistance can result from alterations in FCY2, FCY1 and FUR1, affecting 5FC uptake and its intracellular conversion to active fluorinated metabolites that interfere with RNA/protein and DNA synthesis.

Cryptococcal meningitis has an annual prevalence of 194,000 cases with a mortality of ca. 76% [3]. The primary site of Cryptococcal colonisation is the lung following inhalation of fungal spores, where infection can be asymptomatic and localised or spread invasively and disseminate to the CNS, resulting in meningitis or meningoencephalitis [36]. The treatment of CNS infections is hindered by the inability of antifungal drugs to penetrate the blood–brain barrier (BBB), reducing efficacy [37]. For HIV patients who have CM with innate fluconazole resistance (Figure 1), flucytosine is administered as a combination therapy with AMPB as the gold standard. Unfortunately, this therapeutic is not regularly available in LMICs [38]. In middle-income countries, however, flucytosine-based treatment is routinely applied, allowing for significantly improved survival rates [38]. The mortality of CM in LMIC countries is 22% to 96% at 3 months compared to <15% in Western Europe and North America [24]. AMPB is applied for CM due to its rapid and broad-spectrum activity (Table 2); however, its nephrotoxicity, adverse drug reactions (ADRs) (Table 2) and the increasing emergence of AMPB-resistant or AMPB-tolerant *C. neoformans* impact its clinical efficacy [39]. Table 2 outlines the spectrum of activity, disadvantages and ADRs of the main antifungal agents used in the treatment of IFIs [40], with key resistance mechanisms demonstrated in Figure 1. The studies by Long et al. (2025) identified treatment failure of AMPB combinations with fluconazole/flucytosine against AMPB-resistant *C. neoformans* meningitis [41]. Specifically, in an AMPB-resistant cryptococcal meningitis infection, treatment failure occurred with the administration of AMPB and fluconazole for 14 days followed by fluconazole/flucytosine for 29 days. An antifungal response was achieved using therapy combining amphotericin B colloidal dispersion with flucytosine [41]. Flucytosine applied in combination with AMPB has improved activity against *Cryptococcus* species, resulting in a 20% improved survival rate in CM patients [24].

IA has an annual prevalence of ca. 300,000 cases globally, which is expected to increase due to an increasing population of immunocompromised patients. The annual incidence of IA in ICU patients is ca. 518,960 cases [3], with a mortality rate reaching ca. 88% [20]. *Aspergillus* species also display innate fluconazole resistance and are typically treated with voriconazole, isovuconazole or AMP B [21]. Studies have shown that *A. fumigants* species were present in 92% of IA patients, with *A. niger*, *A. terreus* and *A. flavus* also detected in some cases [42], while IA was detected via autopsy in many undiagnosed ICU patients. The studies by Mudrakola et al. (2022) report that IA was only accurately listed as the cause of death in 27% of cases confirmed via autopsy in patients with typical risk factors for IA or lung disease [20]. The findings of Wang et al. (2024) identified additional risk factors for treatment failure in IA patients, including lower serum albumin, polymicrobial infection, e.g., *P. jirovecii* co-infection [40], elevated procalcitonin (PCT) and less bronchial wall thickening. Surveillance studies show that IA occurred in 0.2% of patients in Italian ICUs, with a mortality of 60%, while an additional study reported a mortality of 91% among 7% of IA patients, many of whom had no predisposing risk factors [31].

Pneumocystis pneumonia (PCP) has an annual prevalence of 505,000 cases and a mortality rate of ca. 46% [3]. The prevalence of PCP in HIV patients reached ca. 80% before the implementation of PCP prophylaxis and antiretroviral therapy [43,44]. There is an increase in the prevalence of *Pneumocystis jirovecii* (PJP) in immunocompromised patients without HIV infection, e.g., autoimmune, haematopoietic stem cell transplantation (HSCT) (CAR-T cells) and hematologic malignancy patients [45]. Mortality is higher in these patients compared to HIV patients [46]. ICU admission and mechanical ventilation negatively impact survival rates in both HIV and non-HIV patients [44]. Trimethoprim–sulfamethoxazole (TMP-SMX) is the recommended prophylactic agent for PCP [47]. The European Crohn’s and Colitis Organisation (ECCO) guidelines suggest the administration of prophylactic TMP-SMX to patients on multiple immunosuppressive therapies, such as steroids, methotrexate, thiopurines, and biologics [48]. Additionally, the European Alliance of Associations for Rheumatology (EULAR) endorses PCP prophylaxis in patients receiving 15–30 mg of prednisolone for ca. 1 month [49]. Importantly, extrapulmonary pneumocystis (EPCP) with infection of the lymph nodes, liver, CNS and thymus has been identified in some patients [50]. Studies determined an overall 6-month mortality rate of 40.5% in non-HIV patients in ICUs, where patients did not receive prophylaxis or received it late [51]. Despite its prevalence and alarming mortality rate, little is known about the exact aetiology of PJP, as it is not currently possible to cultivate *Pneumocystis* in vitro [52].

**Table 2:** Disadvantages of key antifungal agents used in the treatment of IFIs.

Antifungal	Spectrum of Activity	Disadvantage	Reference
Pyrimidines—Flucytosine (5FC)	Fungistatic; limited activity against yeast species, e.g., *Candida* species, *Cryptococcus* species, and certain moulds, not active against *Aspergillus* species, *Mucorales*, or endemic primary pathogens; antimetabolite drug; prodrug.	Renal dysfunction, limited access in LMICs, lower bioavailability among late-stage HIV patients, dose-related toxicity, bone marrow suppression (leukopenia and thrombocytopenia), hepatotoxicity, GIT issues, resistance issues, and teratogenic activity in rat species.	[24,25,29,39]
Polyene—AMPB	Fungicidal, broad-spectrum activity.	Nephrotoxicity, resistant or tolerance detected in *C. neoformans*, and low solubility.	[29,39,53]
Azoles—Fluconazole Voriconazole Isavuconazole Oteseconazole	Fungistatic. Broad spectrum of activity against most *Candida* but not *C. glabrata and C. krusei*; *active against Cryptococcus* and other dimorphic yeast; CNS penetration. Voriconazole is the front-line therapy for invasive aspergillosis; inactive against *Mucorales* spp. Broader spectrum of activity, including *Mucorales*; prodrug. Vulvovaginal candidiasis; active against fluconazole-resistant *C. glabrata*.	Survival of tolerant fungal subpopulations; influenced by genetic polymorphisms in drug-metabolising enzymes. Resistance is common: 90% of *C. auris* are resistance to fluconazole; resistance present in *C. albicans*. Potential liver and kidney issues—requires monitoring for neurotoxic effects, photosensitivity and phototoxic skin reactions. GIT issues; liver toxicity is a risk; drug–drug interactions. Not used in pregnant or breast-feeding women; GIT upset.	[2,11,30,34]
Echinocandins—caspofungin, micafungin, anidulafungin, rezafungin	Fungicidal against *Candida* species; fungistatic against *Aspergillus* species; prophylaxis of *Candida* in HSCT patients.	*Cryptococcus* species are resistant. No EU breakpoints available. GIT issues, hypersensitivity reaction, liver monitoring needed, and IV administration only.	[29,45,54,55]
Antibiotic combination—Trimethoprim–sulfamethoxazole (TMP-SMX)	Prophylactic agent for PCP.	Adverse reactions, e.g., rash bullae, ulcerations, Stevens–Johnson syndrome or toxic epidermal necrolysis.	[47]

### 2.1. Fungal Sepsis

Sepsis remains a great challenge for clinical treatment, with high mortality rates, especially in neonatal and other vulnerable patients. The WHO classifies sepsis as a global health threat, with prevention and treatment being key to the SDG targets, including 3.8 on quality of care, as well as 3.1 and 3.2 on reducing mortality rates in vulnerable populations [56]. The immune response to fungal infection is initiated following recognition of pathogen-associated molecular patterns (PAMPs) present on microbial species by host immune cell pattern recognition receptors (PRRs), e.g., Toll-like receptors (TLRs), C-type lectin (CLEC)-like receptors, nucleotide-binding oligomerisation domain (NOD)-like receptors, and inflammasomes [57]. This interaction triggers a cytokine-mediated pro-inflammatory response and B cell and T cell [58] activation in innate and adaptive immunity, which are overstimulated in sepsis. Sepsis is, therefore, an overstimulation and dysregulation of the immune system (Table 3), leading to a cytokine storm and neutrophil activation, which contributes to organ dysfunction, compensatory anti-inflammatory response syndrome (CARS), persistent inflammation–immune suppression, multi-organ dysfunction syndrome (MODS), and catabolism syndrome (PICS) [59]. *Candida* species-induced immune dysregulation and clinical outcomes in sepsis patients are summarised in Table 3. Candida virulence factors and induced inflammatory responses in vivo are also highlighted. CARS in sepsis patients is associated with secondary infections, high mortality rates, reactivation of latent infections due to a decrease in immune function and increased risk of nosocomial infections [60]. Sepsis has a mortality rate of 40% in ICUs and ca. 25% in general hospitals; additionally, 75% of recovered patients have a higher prevalence of chronic morbidities, including morbidities of the central nervous system (CNS) and post-sepsis syndrome (PSS) [61]. The studies by Zeleke et al. (2025) show a mortality rate of ca. 67% for septic shock in Ethiopia, an LMIC, compared to ca. 45% in France, a developed country [62].

Fungal sepsis has an underreported prevalence, with high mortality rates in at-risk patients, including IC patients, ICU patients (neonatal and adult), patients with indwelling medical devices and co-morbidities of hematologic malignancies, dementia and kidney disease [63]. HSCT, organ transplantation and high-dose corticosteroid use are additional risk factors for fungemia, which is considered a precursor to sepsis [64]. Recent studies report that the most common fungal species associated with sepsis are *Candida albicans*, followed by non-albicans *Candida* species, *P. jirovecii* and *Aspergillus* species [45]. Neonatal ICU surveillance shows that *Candida* species are causative of high rates of late-onset sepsis and mortality in low-birth-weight and preterm infants [65,66]. Studies show that ca. 30% of inflammatory bowel disease (IBD) patients develop sepsis following central venous catheter placement, with 20% of these sepsis cases due to *Candida* species [48]. The studies by Shin et al. (2023) describe a case of an *Aspergillus* IFI and subsequent septic shock in an elderly patient with no established risk factors [67]. The case report by Simonetti et al. (2024) describes *Cryptococcus* septic shock in an elderly non-HIV patient on prolonged steroid treatment [68]. Another study describes the death of an immunocompetent patient due to septic shock and multiple organ dysfunction due to co-infection with *Neisseria meningitidis* and *C. neoformans* [69]. Importantly, *Candida* BSIs are common polymicrobial infections by bacterial species, including coagulase-negative *Staphylococcus*, *Klebsiella pneumoniae*, and *Staphylococcus aureus* [70]. Toritani et al. (2025) highlight the difficulty of diagnosing a PJP infection in a patient with *Candida* sepsis [48]. The case describes an immunosuppressed elderly patient with IBD who had severe PJP with postoperative *Candida* sepsis, in whom PJP was difficult to diagnose [48].

The use of empiric antifungal therapy in fungemia is contradictory. The Surviving Sepsis Campaign 2021 states that the administration of antifungal therapy must depend on variable risk factors, citing studies showing no difference in mortality rates between cohorts receiving antifungal therapy and a placebo [64]. Importantly, studies show that the prophylactic use of fluconazole antifungal therapy for invasive *Candida* infections may contribute to patient survival, especially in adult allogeneic HSCT patients [71]. Such prophylaxis, however, is believed to contribute to the emergence of fluconazole resistance in *C. albicans* and non-*albicans* species (*C. glabrata*, *C. krusei*) [72].

**Table 3:** *Candida* species-induced immune dysregulation and clinical outcomes in sepsis patients.

Fungal Virulence Factor	Immune Component	Dysregulation	Clinical Effect	Reference
*Candida’s* cell wall—mannose and glucan PAMPs [59]	CD4+ T cells—T-helper 1 (Th1), T-helper 2, T-helper 17 (Th17)	Interleukin-17 (IL-17)	Recognised by host PRRs—immune stimulation	[73]
	Regulatory B cells	IL-10 production	Persistent inflammation	
Chitin from *Candida albicans*	Cytokines, e.g., IL-10	Pro- and anti-inflammatory	Persistent inflammation—cytokine storm–refractory fever, cytopenia, coagulopathy, MODS, mortality	
Pyroptosis by *Candida*	Macrophage destruction—inflammatory programmed cell death	Inactivation of immune response	Survival of phagocytosis and innate immunity	[57]
Dimorphic nature of *Candida* species	Fungal wall zymosan activates B and T cells	IFIs	Disseminated infection, systemic mycosis, sepsis	

### 2.2. Virulence Factors Promoting Pathogenicity

Fungal virulence factors promote pathogenesis via immune evasion, tissue invasion and disseminated infection. To cause human infection, yeast and fungal species must tolerate and proliferate at 37 °C, which limits IFIs to certain species, including the clinically relevant *Candida*, *Aspergillus*, *Cryptococcus* and *Pneumocystis*. As pathobionts, yeast-like species in the genus *Candida* are associated with surgical procedures, which allow systemic movement from the GIT [13], and/or the placement of indwelling medical devices, which break the skin barrier. Importantly, the dimorphic nature of *Candida* species, with cells shifting from unicellular yeast to filamentous hyphae, allows for IFIs [74]. Exogenous fungal pathogens, including *Aspergillus*, *Cryptococcus* and *Pneumocystis,* produce airborne spores that are inhaled, allowing for respiratory colonisation and subsequent systemic dissemination [9]. The host immune system in healthy people can typically eliminate fungal colonisers, whereas in IC patients, reduced immune activity allows for fungal dominance [75]. The presence of virulence factors in pathogenic fungi leads to pathogenesis in immunocompetent persons (Table 4). The main virulence factors that promote the pathogenicity of *Aspergillus*, *Candida*, *Cryptococcus*, and *Pneumocystis* are provided in Table 4. To establish pathogenesis, fungal cells must be able to evade the host immune system, which mounts an aggressive humoral and cell-mediated attack. Their genetic and morphological plasticity allows for metabolic adaptation to the host environment, tissue lysis and nutrient absorption, evasion of macrophage phagocytosis and host antimicrobial action. *C. neoformans* is a facultative intracellular pathogen that colonises host macrophages and replicates in tissues [39]. *C. neoformans* also produces a robust capsule, which confers protection against oxidative stress in vivo and prevents recognition by cell-mediated immune response [76]. *Aspergillus* species rapidly form hyphae that invade blood vessels and disseminate to bodily organs [10]. *Aspergillus* species also produce toxins with immunosuppressive action, e.g., gliotoxin, and toxins associated with hypersensitivity, e.g., aflatoxins and ochratoxin A [77]. Gliotoxin is an important virulence factor of *A. fumigatus,* as it inhibits the host’s immune response, enables tissue invasion, and may induce cell apoptosis [78]. *C. albicans* can perform induced endocytosis using invasions present on hyphae, which stimulate pseudopod production by host cells and active penetration [74]. *C. albicans* hyphae and cell wall components, e.g., Zymosan, activate B cells typically via TLRs, which promotes the production of antibodies (Table 4), the release of IL-6 and T cell activation [73]. *C. auris* has high osmotolerance, halotolerance and thermotolerance, allowing for survival on skin and disinfectant resistance [79]. Importantly, *C. auris* does not form hyphae or pseudohyphae and grows at 40–42 °C [80]. The production of adhesins by fungal cells, which bind to host cell surface proteins and promote attachment and invasion, is an important virulence factor. As is the secretion of enzymes such as lipases, proteases and nucleases, which enable nutrient uptake, host cell cytotoxicity and tissue damage [81]. These degradative enzymes allow *C. neoformans*, *Pneumocystis*, *A. fumigatus*, *C. albicans*, and *C. auris* to disseminate in vivo, avoiding host immunity [81]. Heat shock proteins (HSPs) improve environmental stress tolerance in fungal species, e.g., heat stress, antimicrobial agents, and immune attack, and are key to fungal pathogenicity, with HSP110, HSP104, HSP90 and HSP 70 present in *Candida*, *Cryptococcus* and *Aspergillus* species [82]. HSP90 is involved in conferring antifungal resistance in pathogenic fungi. An excellent review of HSPs in fungal species is provided by Padró-Villegas et al. (2026) [82]. *C. albicans* can cause pyroptosis, damaging macrophages, which acts as a survival mechanism in vivo [59]. Pyroptosis is associated with the dimorphic nature of *Candida*; it triggers inflammatory apoptosis via an inflammasome protein complex and caspase-1 in macrophage cells, ultimately resulting in the formation of pores in the macrophage cell membrane [83]. Uninhibited inflammasome activation can promote inflammation and tissue damage in vivo [84]. Pyroptosis is, therefore, a potent virulence factor of *Candida* species in the aetiology of fungaemia and sepsis (Table 4). Such immune regulatory dysregulation and disturbance is associated with immune paralysis, which suppresses the immune response and supports pathogenicity [31].

Yeast and fungi are prolific biofilm producers, which enables attachment to host tissue surfaces in vivo, where the morphological switch between yeast and hyphae structures enhances immune evasion and pathogenicity. The studies by Honorato et al. (2022) show that fungal extracellular vesicles (EVs) transmit molecules, including diterpenes and fatty acids, which influence biofilm formation, morphology and virulence in *C. albicans* [85]. EVs typically contain many biologically active compounds, such as nucleic acids, carbohydrates, and enzymes. *C. neoformans* EVs, for example, contain podoconjugates, which are important virulence factors [86]. *A. fumigatus* EVs mediate β-glucan-stimulated IL-1α secretion by neutrophils, with a pro-inflammatory action [87]. *C. neoformans* and *A. fumigatus* EVs contain polysaccharide and hydrophobic protein structures, preventing macrophage phagocytosis and reducing innate immune activation [88]. *Cryptococcal* EVs are important for capsule formation and maintenance, as they transport glucuronoxylomannan (GXM), which is essential for capsule structure [89]. Furthermore, GXM binds immune complement proteins, inhibiting opsonisation and phagocytosis by host macrophages and preventing an effective immune response, thereby ensuring fungal survival in vivo [90]. EVs of *C. auris* impact host cell immunity, epithelial cell adhesion and macrophage action, promoting fungal cell survival [91]. HSP 70 is believed to contribute to biofilm formation in *C. neoformans*, *A. fumigatus*, and *C. albicans* [92]. Adhesin proteins, e.g., *ALS1*, *ALS2*, *ALS4*, are important for fungal biofilm formation and persistence [54]. Biofilms in yeast and mould fungi typically exhibit polymorphological structures, which hinders therapeutic and immunological targeting [93]. The formation of Cryptococcal biofilms in the CNS or brainstem (cryptococcoma) of CM patients has been misdiagnosed as malignancy or brain tumours [94]. Sayson et al. (2024) theorise that *Pneumocystis* can use extracellular DNA in biofilm formation as a scaffold, similar to the actions of *C. albicans* and *A. fumigatus* [84]. Gliotoxin is an important secondary metabolite produced by *Aspergillus* biofilms, which has potent virulence [78]. Additionally, *A. fumigatus* biofilms possess anoxic regions that are involved in cell quiescence and drug resistance [95]. Studies show that fungal biofilms display ca. 2000 times more AFR compared to their planktonic counterparts due to reduced drug penetration, altered metabolic activity, and the upregulation of fungal efflux pumps and AFR genes [96]. The treatment of fungal biofilms, therefore, requires higher antifungal dosage concentrations, which raises issues of biocompatibility and toxicity in vivo and/or the removal of medical devices [29]. Importantly, a clinical laboratory’s inability to differentiate between planktonic and biofilm fungal infections impacts diagnostic and treatment protocols [18]. Undoubtedly, fungal biofilm formation in vivo increases the morbidity and mortality rates associated with IFIs [29]. The studies by Atiencia-Carrera et al. (2022) show that the mortality rate of *Candida* BSIs was 70% when a biofilm was present, compared to ca. 38% in the absence of a biofilm [18]. The findings of Martins et al. (2026) demonstrate that *Candida* biofilm formation negatively impacts catheter BSI, therapeutic and patient outcomes in neonatal ICU patients [97].

**Table 4:** Fungal virulence factors promoting pathogenicity.

Virulence Factor	Pathogenicity	Species	Reference
Spore production	Airborne—respiration inhalation with local and possible systemic involvement	*Aspergillus*, *Cryptococcus*, *Pneumocystis*	[9,36]
Toxin production Candidalysin Phospholipase B Gliotoxin Aflatoxins and ochratoxin A	Pore-forming peptides Lipolytic activities Immunosuppressive toxins Hypersensitivity reactions	*C. albicans* *Aspergillus* *A. fumigatus* *A. fumigatus*	[77]
AMR/MDR Plasmids	Resistance to antifungal agents, resistance to biocidal agents MDR transfer	*Aspergillus*, *Cryptococcus*, *Pneumocystis*, *Candida species*	[29]
Biofilm formation	Medical device colonisation, e.g., catheters, colonisation in vivo, increased AFR, environmental adaptation	*Candida*, *Aspergillus*, *Cryptococcus*, *Pneumocystis*	[78,96]
Osmotolerance (>10% NaCl) Halotolerance	Biocidal resistance Survival on skin	*C. auris*	[79]
Genetic and morphologic plasticity	Readily adapt to changing environmental conditions	*Candida*, *Aspergillus*, *Cryptococcus*, *Pneumocystis*	[39]
Capsule formation	Immune evasion	*Cryptococcus*	[76,89]
Melanin production	UV resistance; adaption to desiccation, oxygen radicals, and extreme temperatures	*Aspergillus*, *Cryptococcus*, *C. albicans*, *C. auris*, *C. glabrata*, *Pneumocystis*	[98]
Active penetration and induced endocytosis	Intracellular colonisation/invasion	*C. albicans*	[74]
Dimorphism	IFIs, sepsis, immune evasion	*C. albicans*, *C. neoformans*	[31]
Pyroptosis	Immune paralysis, IFI, sepsis	*C. albicans*	
Extracellular vesicles (EVs)	Biofilm formation, cell communication, adhesion, anti-phagocytotic activity β-glucan-stimulated Capsule formation	*Candida species* *Cryptococcus* *A*. *fumigatus* *C. neoformans*	[88,99]

## 3. Novel Approaches in Fungal Treatment

With increasing rates of AFR, poor bioavailability, drug-induced toxicity and lack of availability in LMICs, there is an urgent need for novel antifungal compounds that are safe, potent and affordable. Current research is focused on finding new antifungal targets, identifying antifungal drug combinations, repurposing other therapeutics as antifungal agents, and developing advanced delivery systems, e.g., nanoparticles and liposomes, to improve bioavailability and BBB penetration [37]. Combination therapy has proven effective in the treatment of CM. For example, AMPB acts on ergosterol in the cell membrane when combined with flucytosine, which inhibits nucleic acid synthesis [38], and with fluconazole, which inhibits ergosterol synthesis and is more readily available in LMICs. Additionally, combination with therapeutics such as artemisinin (ART) and antibacterial agents such as TMP-SMX has shown efficacy in treating IFIs, including PCP [48]. More recently, the studies by Chakraborty et al. (2025) demonstrated the efficacy of combining the antibacterial besifloxacin with fluconazole, achieving a greatly reduced MIC for fluconazole (2 to 0.5 mg L^−1^) and clearing kidney infections by 83% in test mice [100]. Importantly, the anticancer agent Panobinostat reduced the MIC of fluconazole (128 to 0.5–2 μg mL^−1^) in azole-resistant *C. albicans* and demonstrated antibiofilm activity [101]. De Oliveira et al. (2026) investigated the activity of 2-mercaptobenzothiazole (MBT) in combination with sulfonamides against MDR *C. albicans,* demonstrating resistance to many azoles and anidulafungin [102]. Pitavastatin calcium demonstrated a synergistic effect with fluconazole against *C. albicans* at low concentrations, preventing toxicity and adverse effects [103].

Tetrazoles are new antifungal agents that inhibit fungal growth by targeting the CYP51 enzyme and include oteseconazole [30]. The triazole isavuconazonium sulfate (prodrug of the triazole isavuconazole) demonstrates efficacy against IA and many yeasts, moulds, and dimorphic fungi, including *C. albicans* and *Cryptococcus*, with an improved biocompatibility profile [104]. The studies by Zhang et al. (2025) describe the broad-spectrum antifungal action of a novel tetrazole derivative called T24, which has antibiofilm activity [105]. T24 also appears capable of inhibiting the transition of yeast cells to fungal cells, which is important for invasive mycosis [105]. Polyenes, including AMPB, natamycin and nystatin, are the most potent antifungals clinically, but they are limited by their toxicity profiles. Mirza et al. (2026) describe an improved antifungal action against IA and reduced in vivo toxicity of Nys34, a novel polyene compound [53]. Rezafungin, approved by the FDA in 2023, is a novel echinocandin antifungal with an improved safety profile and efficacy against *Candida* species, including *C. auris* [71]. Fosmanogepix is currently in Phase 3 clinical trials for the treatment of invasive Candidiasis as oral and intravenous (IV) formulations (NCT05421858). Olorofim is in Phase 3 trials for the treatment of IA (NCT05101187), while the Phase III trial for opelconazole in the treatment of pulmonary aspergillosis was terminated in 2026 (NCT05037851). The WHO “Antifungal agents in clinical and preclinical development” 2025 report outlines novel antifungal agents and current therapeutics that may be repurposed as antifungal agents, such as tamoxifen, clindamycin, sertraline and panobinostat [106]. The report shows that there are potential therapeutics in the preclinical pipeline for all WHO priority pathogens, with nine compounds demonstrating activity against critically important fungi, including *Aspergillus*, *Candida* and *Cryptococcus* [106].

Antimicrobial peptides (AMPs) have demonstrated efficacy in the control of AMR pathogens. Additionally, AMPs have many advantages, including ease of production, biocompatibility, immune regulatory functions, and potent and rapid action [107]. The identification of antifungal AMPs with broad-spectrum activity against MDR fungi may provide novel therapeutic options. The studies by Zhou et al. (2024) investigate the multi-target activity of (Gly_0.8_Nap_0.2_)_20_ against MDR *Candida*, *Cryptococcus* and *Aspergillus* species, with no observed resistance evident [108]. The AMP PmUreβ was active against biofilms of *C. albicans* and proved effective against *C. auris* when combined with fluconazole [109]. Roque-Borda et al. (2025) provide an excellent review of AMPs as novel antifungal agents [110]. Mycoviruses, which are viruses that specifically infect fungi, also show potential as antifungal agents. The study by Hasan et al. (2025) describes the isolation and characterisation of a phage with activity against a reference MDR ATCC strain of *C. albicans* (ATCC 10231) [111], which is not a clinical isolate. Such studies need to be conducted on clinical isolates to determine their therapeutic efficacy against IFIs. Initial studies show that mycoviruses can disrupt fungal biofilm formation and hyphae production. Viruses are also amenable to genetic modification (GM) and can be engineered to act as immune-boosting adjuvants [112]. The role of mycoviruses in promoting fungal virulence, pathogenicity, adaptation and fitness, however, is a concern for their application [113], as well as their isolation, formulation, stability and regulatory limitations [114].

### Future Directions in the Treatment of Invasive Fungal Diseases

The application of nanoparticles and nanotechnology in the treatment of AMR infectious diseases may allow for enhanced efficacy, improved biocompatibility and reduced mortality rates. Nanoparticles (NPs) have been applied as drug delivery systems, allowing for improved solubility, more targeted action, biofilm penetration and reduced systemic toxicity [115]. There are three categories of medical NPs, including organic NPs (polymers, liposomes, dendrimers), which are nontoxic and biodegradable; inorganic NPs based on metals and metal oxides; and polymeric NPs. Lipid NPs have many advantages over polymer NPs, such as improved biocompatibility, cheaper production, scale-up and tolerability in vivo [116]. The studies by El-Gazzar et al. (2024) assessed the efficacy of zinc NPs (ZnNPs) and nanocomposites (ZnNCs) against *C. albicans* isolates, which had antifungal and antibiofilm action [117]. Biogenic silver NPs (AgNPs) in combination with antifungal drugs, including itraconazole, terbinafine, and clotrimazole, demonstrated antifungal activity against MDR *Candida species* [118]. NP drug delivery systems offer advantages for pulmonary aspergillosis, as demonstrated by Jeong et al. (2025), who developed an inhalable itraconazole formulation within a polyvinyl alcohol nanoparticle-in-microparticle (NIM) system [119]. This delivery method demonstrated greater drug targeting while reducing systemic toxicity in vivo [119]. Li et al. (2025) developed posaconazole nanocrystal-agglomerated particles for dry powder inhalers (DPIs) for the treatment of pulmonary aspergillosis, which showed improved solubility and dissolution rates with limited cell toxicity [120]. Fayyaz et al. (2024) developed a DPPC/DMPG surface-modified compritol lipid NP carrying voriconazole for pulmonary aspergillosis in IC patients [116]. The research by Lanser et al. (2026) investigated a poly(D,L-lactide-co-glycolide) NP with the fungal metalloprotease Mpr1 and encapsulated AMPB, which demonstrated increased BBB penetration in the treatment of *C. neoformans* [121]. While nanotechnology-based systems have clear potential, they are hindered by biocompatibility issues *in vivo*, a lack of pharmacokinetic information, production issues and a lack of harmonisation in manufacturing methods [122]. Bhardwaj et al. (2025) provide an excellent account of protein-based NP drug delivery methods [123].

Research has highlighted the importance of fungal EVs in the aetiology of mycosis [85]. EVs may offer diagnostic aids in IFIs by acting as biomarkers and therapeutic targets. The research by de Rezende et al. (2024) identified the presence of fungal EVs in serum and urine samples from patients with candidiasis and cryptococcosis and suggested their potential in the treatment of systemic mycoses [124]. Fungal EVs may also be bioengineered to act as drug delivery vehicles that can bypass biological barriers in vivo [125]. EVs may be suitable vaccine platforms due to their PAMPs and fungal antigen components, as demonstrated in a mouse model against *A. fumigatus* [99]. Alternatively, targeting and inhibiting fungal EVs to prevent cell communication and biofilm formation in vivo may offer therapeutic options. According to the studies by Kim et al. (2025), drugs that inhibit exosome formation are effective against *Candida* EVs by impacting biofilm production and increasing cell susceptibility to fluconazole [126]. Drugs that inhibit HSPs also show potential as antifungal agents or adjuvants for use in combination with antifungal drugs. The antibiotic geldanamycin, for example, was found to inhibit the movement of HSP90 into mitochondria and enable azole toxicity against *C. albicans* [127]. Drugs targeting HSPs are problematic, however, as side effects, including liver, cardio, GIT and neuron toxicity, are high risk [128]. Targeting HSPs in the treatment of mycosis is challenging because fungal HSPs have similarities to mammalian HSPs [82]. This may be overcome, however, by targeting extracellular HSPs [129]. The inhibition of HSP90 in *C. neoformans* increased its susceptibility to fluconazole and conferred fungicidal action against *C. auris* and *C. albicans* [130]. Padró-Villegas et al. (2026) provide an excellent account of HSPs as potential antifungal agents [82].

Nucleic acid (NA) therapies have emerged as novel therapeutic options in the treatment of infectious diseases. NA therapies include varying forms of DNA and RNA moieties, which are categorised based on their mode of action as gene silencing, protein inhibition, gene editing, and gene expression NAs [131]. In treating infectious diseases, NA therapy aims to provide a form of RNA (mRNA, siRNA, anti-miR, ASO, aptamers) to downregulate genes, induce gene overexpression or deliver PRRs that trigger immune responses [132]. Clustered Regularly Interspaced Short Palindromic Repeats (CRISPR/Cas) technology is showing promise as a gene editing tool and a diagnostic aid in IFIs [133]. Mundhe and Rajkumari (2025) describe the application of RNA interference (RNAi) as a novel RNAi-based gene silencing tool against MDR *C. albicans* [134]. The studies by Li et al. (2025) developed an effective lipid NP mRNA vaccine for *Cryptococcus* with a capsule adjuvant, which showed efficacy in test mice [135]. Unfortunately, the application of NA technology for IFDs is hindered by many issues, including NA degradation in vivo, stability and storage, cost of production, optimal RNA targets, and passage through the fungal cell wall [136]. Additionally, the application of immune-boosting NA technology in IFD patients, who are predominantly IC, poses challenges [135]. As does the application of immunotherapy therapeutics, such as immune checkpoint inhibitors and cytokine and antibody-based therapies, due to the increased risk of iatrogenic immune issues in patients with pre-existing immune dysregulation [137]. Advances in NA technologies, such as sequencing technologies, have occurred rapidly, allowing for the identification of genetic, protein and additional markers in infectious diseases. When coupled with advances in mass spectrometry and electron microscopy, these technologies may allow for more targeted and potent antifungal options.

Advancements in the clinical treatment of IFDs may be achieved with the application of Quantitative Structure–Activity Relationship (QSAR) models, microfluidic technology (MF), machine learning (ML), digital twins, and artificial intelligence (AI). Kaushik et al. (2026) applied a QSAR model to identify novel CYP51 inhibitors to circumvent azole resistance in fungal species [138], similar to Salehi et al. (2022), who applied QSAR and molecular modelling techniques to assess the antifungal potential of fluconazole-like structures [139]. The studies by Zapadka et al. (2026) applied a QSAR analysis model using genetic algorithms and multiple linear regression to identify compounds with antifungal activity [140]. ML has also been employed to identify key features of antifungal polymers [141] and new antifungal molecules against *C. albicans* [142]. The studies by Duroux et al. (2026) applied an ML pipeline integrating MALDI-TOF mass spectrometry to predict AFR in *C. albicans* with micafungin resistance and *C. parapsilosis* with fluconazole resistance [143]. Recently, an AI/ML approach was applied to identify antifungal peptides with activity against *C. albicans* but not *C. auris*, with low hepatotoxicity *in vitro* [144]. ML and AI tools are limited by a lack of high-quality fungal datasets and specific fungal genomic information. This may be improved by the application of newer generative AI models, including protein diffusion models and small molecule diffusion models [144]. Zhang et al. (2026) applied whole-genome sequencing to determine AMR in clinical *Candida* species using large-scale population genomic analysis of environmental and clinical isolates [145]. The application of such techniques contributes to Quality by Design (QbD) in drug development, as sought by the European Medicines Agency (EMA). Quality by Design aims to “ensure the quality of medicines by employing statistical, analytical and risk-management methodology in the design, development and manufacturing of medicines” [146]. Mahajan et al. (2025) applied a QbD approach to develop an ultra-deformable ethosome (UDE)-based nanocarrier for luliconazole in the treatment of skin fungal infections [147]. Roopesh and Chandramouli (2019) applied a QbD approach to optimise critical quality attributes in the design of an antifungal formulation of itraconazole [148]. Studies demonstrate that a QbD-optimised SER-NEG antifungal system had improved stability, controlled drug release, and more potent action against *Candida* and *Aspergillus* species [149]. A Film-Forming Gel (FFG) containing itraconazole was developed by Somwanshi and Donde (2025) using a QbD approach, which had improved diffusion, increased antifungal efficacy against *C. albicans*, and formulation stability [150]. The advantages of the QbD approach are outlined by Duarte et al. (2025) [151]. These formulations demonstrate the importance of QbD in the development of antifungal agents for dermal fungal infections but not IFIs, and they do not demonstrate direct clinical evidence in IFIs. QbD should, therefore, be considered in the formulation of therapeutics for oral and respiratory drug delivery in the treatment of IFIs. The development of effective fungal vaccines is an ongoing area of research, where advances in immunotherapy may offer solutions [152]. An excellent review is provided by Inácio et al. (2023) [152]. Issues remain, however, related to large population sizes for clinical trials, similarity to human eukaryotic cells and the cost of large-scale production. Currently, three vaccines, namely, NDV-3A, PEV7, and Candi5V, are in clinical trials for recurrent vulvovaginal candidiasis [153]. EVs may also offer beneficial vaccine modalities, as studies show that *C. albicans* EVs increased IgG antibody concentrations in vaccinated mice and reduced fungal load [154].

## 4. Conclusions

Invasive fungal diseases represent a significant health risk, with increasing rates of morbidity and mortality. Candidiasis, aspergillosis, cryptococcosis, and pneumocystis are responsible for ca. 90% of reported invasive fungal deaths. Fungal infections are often chronic, persisting for extended periods, and recurrent, particularly in IC patients. Fungal species possess many virulence factors that promote their pathogenicity. Antifungal resistance is a major driver of mortality in immunocompromised patients. Increasing resistance to antifungals, including azoles, polyenes and echinocandins, is due to the overexpression of efflux pumps, mutations in the CYP51 gene and alteration of ergosterol biosynthesis. Isavuconazole sulfate, a triazole prodrug, and the tetrazole oteseconazole, along with the echinocandin rezafungin, are new antifungal agents that act to inhibit fungal growth. Tetrazoles act by targeting CYP51, demonstrating efficacy against IA, *C. albicans*, and *Cryptococcus,* with an improved biocompatibility profile. Isavuconazole inhibits cytochrome P450-dependent lanosterol 14α-demethylase, while Rezafungin is a β-glucan synthase inhibitor. Additionally, traditional antifungal therapeutics may be successfully augmented using other therapeutics such as artemisinin, TMP-SMX and Panobinostat. Such combinations, however, do not improve the biocompatibility profile of current antifungal agents. Advances in nucleic acid technology and nanoparticle delivery systems may offer alternative or improved antifungal options. The application of mRNA formulations has grown quickly since the COVID-19 pandemic and may offer vaccine solutions for IFDs. While limitations remain, access to such options in LMICs is an important factor, as developing countries suffer greater rates of fungal mortality with limited access to treatment. Novel methods using genomics, ML and AI also allow for the identification of drug targets, resistance genes and potential biomarkers for fungal disease. Undoubtedly, the clinical treatment of IFDs is undergoing a transformation driven by advances in these technologies and an increasing understanding of the aetiology of fungal disease and the role of immune dysregulation. At present, however, such applications remain hindered by a lack of relevant datasets and harmonised models. When combating AFR, improved diagnostic protocols, the implementation of stewardship programmes, and increased surveillance are also needed. From a One Health perspective, the use of antifungal agents in agriculture and its relationship to AMR must also be addressed.

## Data Availability

No new data were created or analyzed in this study. Data sharing is not applicable to this article.
